# Cohesin Interaction with Centromeric Minichromosomes Shows a Multi-Complex Rod-Shaped Structure

**DOI:** 10.1371/journal.pone.0002453

**Published:** 2008-06-11

**Authors:** Alexandra Surcel, Douglas Koshland, Hong Ma, Robert T. Simpson

**Affiliations:** 1 The Intercollege Graduate Program in Cell and Developmental Biology, The Huck Institutes of the Life Sciences, Pennsylvania State University, University Park, Pennsylvania, United States of America; 2 Howard Hughes Medical Institute, Department of Embryology, Carnegie Institution of Washington, Baltimore, Maryland, United States of America; 3 Department of Biology, The Pennsylvania State University, University Park, Pennsylvania, United States of America; 4 Department of Biochemistry and Molecular Biology, Pennsylvania State University, University Park, Pennsylvania, United States of America; Duke University, United States of America

## Abstract

Cohesin is the protein complex responsible for maintaining sister chromatid cohesion. Cohesin interacts with centromeres and specific loci along chromosome arms known as Chromosome Attachment Regions (CARs). The cohesin holocomplex contains four subunits. Two of them, Smc1p (Structural maintenance of chromosome 1 protein) and Smc3p, are long coiled-coil proteins, which heterodimerize with each other at one end. They are joined together at the other end by a third subunit, Scc1p, which also binds to the fourth subunit, Scc3p. How cohesin interacts with chromosomes is not known, although several models have been proposed, in part on the basis of *in vitro* assembly of purified cohesin proteins. To be able to observe *in vivo* cohesin-chromatin interactions, we have modified a Minichromosome Affinity Purification (MAP) method to isolate a CAR-containing centromeric minichromosome attached to *in vivo* assembled cohesin. Transmission Electron Microscopy (TEM) analysis of these minichromosomes suggests that cohesin assumes a rod shape and interacts with replicated minichromosome at one end of that rod. Additionally, our data implies that more than one cohesin molecule interacts with each pair of replicated minichromsomes. These molecules seem to be packed into a single thick rod, suggesting that the Smc1p and Smc3p subunits may interact extensively.

## Introduction

Proper chromosome segregation is essential for the completion of the mitotic cell cycle and consequently is vital for the development and propagation of living organisms. Failure of sister chromatids to segregate correctly can lead to aneuploidy causing cellular dysfunction and cell death, as well as disorders such as Cornelia de Lange Syndrome (characterized by multiple congenital anomalies) and trisomy 21 or Down's Syndrome [1]–[6]. To ensure that each daughter cell has a complete set of chromosomes, eukaryotic cells guard against aneuploidy by keeping replicated sister chromatids together both at their centromere and along their arms, starting in S phase until they separate at the metaphase-anaphase transition in mitosis. This evolutionarily conserved process, known as Sister Chromatid Cohesion (SCC), is required for the correct attachment by sister kinetochores to microtubules emanating from opposite poles of the spindle and it is believed to establish the tension required to stabilize microtubule-kinetochore attachment [7].

The multimeric protein complex that facilitates SCC is known as cohesin, which is composed of four proteins, Smc1, Smc3, Scc1, and Scc3 [8]–[10]. Two of these – Smc1 and Smc3 – are members of the Structural Maintenance of Chromosome family [8]. Members of this family of proteins are characterized by globular end domains separated by two long coiled-coil arms that are joined together by a flexible, central hinge domain. The hinge domain bends, facilitating the intramolecular anti-parallel interaction between the coiled-coil arms and bringing the two globular domains together to form a functional ATPase of the ABC family of ATPases [11], [12]. Eukaryotic SMC proteins have been shown to form heterodimers mediated by the hinge region [12].

**Figure 1:**
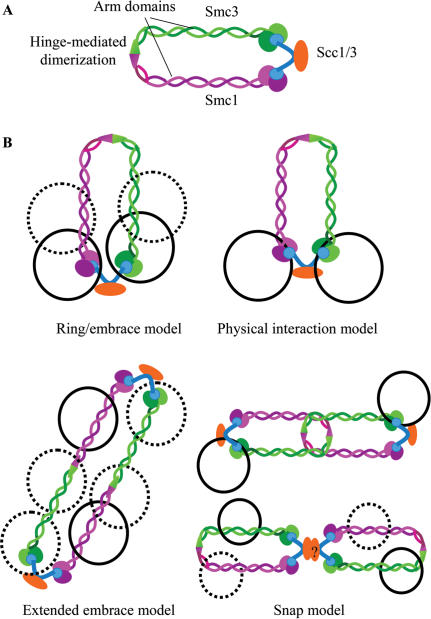
Cohesin models. (A) Schematic of the cohesin complex shows Smc1 (purple) and Smc3 (green) folded onto each other at their respective hinge regions, bringing their terminal domains in close proximity to each other. Dimerization of Smc1 and Smc3 occurs at the hinge domain and the ascribed ring structure of cohesin occurs upon binding of Scc1 to the head domains of Smc1 and Smc3. Scc3 interacts with Scc1 to stabilize the complex. (B) Expected structures of cohesin-bound minichromosomes based on published models. In the embrace model and the extended embrace model, the replicated minichromosomes (black circles) or sister chromatids would be randomly distributed (dotted black circles) along the circumference of one or two cohesin rings respectively. In the physical embrace model, the replicated minichromosomes would be situated at one end of the cohesin ring. The snap model suggests that either the replicated minichromsomes would be randomly distributed along the circumference of two cohesin rings (thus rendering it indistinguishable from the extended embrace model) or maintained at a distance of two cohesin lengths from each other.

Purified recombinant Smc1 and Smc3 can heterodimerize *in vitro* [12]. Rotary shadow TEM imaging revealed that the *in vitro* assembled Smc1/Smc3 heterodimer is a V-shaped molecule with several bends along the length of the arms [12], [13]. Biochemical experiments support the hypothesis that the globular domains of both Smc1 and Smc3 interact with Scc1, which appears to stabilize the interaction of the Smc1/Smc3 heads, resulting in a topological ring (Figure 1A) [12], [14]. However, recent FRET (Fluorescence Resonance Energy Transfer) analyses suggest that Smc1 and Smc3 heads can interact directly without Scc1 [15]. Scc1 in turn interacts with the fourth member of the cohesin holocomplex, Scc3 [12], [13]. The proposed cohesin ring structure is also consistent with TEM images of purified human cohesin complexes [13]. However, these studies do not address possible *in vivo* interactions of the coiled-coil regions of Smc1 and Smc3, similar to those seen for the condensin heterodimer of Smc2 and Smc4 [13].

The cohesin complex binds both at the centromeres and along chromosome arms at specific sites identified in budding yeast as Cohesin Attachment Regions (CARs) [16], [17]. These sites are spaced approximately 9kb apart and are 0.8kb–1.2kb in length. Although CARs do not share any sequence similarity, they are A-T rich and found primarily in intergenic regions. The lack of obvious CAR consensus sequences suggests that cohesin does not interact with chromatin as a sequence-specific DNA-binding protein complex. This lack of specificity, the need to constrain two sister chromatids, and the hypothesized tripartite ring structure of cohesin, have been the primary motivators for the proposal of several models for cohesin binding (Figure 1B). In the ring or embrace model, two 10-nm chromatids (DNA plus histones) are trapped within the cohesin ring and the strength of this topological interaction is sufficient for maintaining SCC. Here, the cohesin ring is hypothesized to open upon ATP hydrolysis, allowing the loading of cohesin onto the unreplicated chromosome. The ring is then proposed to close upon the subsequent binding of another ATP molecule. This model precludes a stable and direct interaction between cohesin and chromatin at CAR loci and fails to explain the continuous maintenance of binding at CARs, each spanning 800 bp or longer.

Two other models exist that are variations of the ring model. In the extended embrace model, two or more cohesin holocomplexes interact to form a larger ring surrounding the two sister chromatids. This larger ring is thought to be formed by the interaction of Scc1p with the Smc1p head of one cohesin molecule and the Smc3p head of a second cohesin molecule. Currently, no evidence for this model exists. The second model that invokes part of the embrace model is the snap model, which suggests that individual sister chromatids are trapped inside separate cohesin rings that are interconnected or bound by as yet unidentified protein partners [18]. Alternatively, the two cohesin molecules in the snap model can also interact directly with chromatin. The fourth model is referred to as the physical interaction model and posits that one cohesin complex interacts with two sister chromatids via the Smc1 and Smc3 heads. There is evidence that condensin interacts with chromosomes by the winding of the chromosome around both the Smc2 and Smc4 heads [19], [20]. Phylogenetic analysis of the SMC proteins suggests that Smc1 is most similar to Smc4 and that Smc3 is most similar to Smc2 [21]. By analogy, cohesin might interact with chromosome via the Smc1 and Smc3 heads, similar to the interaction of the condensin complex with chromatin.

While most of these models have some *in vitro* support, studies of the cohesin holocomplex interacting with sister chromatids have not been performed to validate or refute these hypotheses. Even more importantly, no structural information is available on direct observations of *in vivo* assembled cohesin-chromatin complexes. Understanding how cohesin interacts with DNA or chromatin can shed light not only on how sister chromatid cohesion is maintained, but it may also illuminate how other members of the SMC family of proteins are involved in chromosome condensation and DNA repair function. Standard localization methods such as immunofluorescence do not provide high enough resolution for resolving the competing models for cohesin binding. A method that images either *in vivo* cohesin binding or *in vivo* assembled cohesin-DNA/chromatin complexes such as the Minichromosome Affinity Purification (MAP) method has such a potential.

## Results and Discussion

### Using MAP to isolate minichromosomes and in vivo assembled chromatin-cohesin complexes

**Figure 2:**
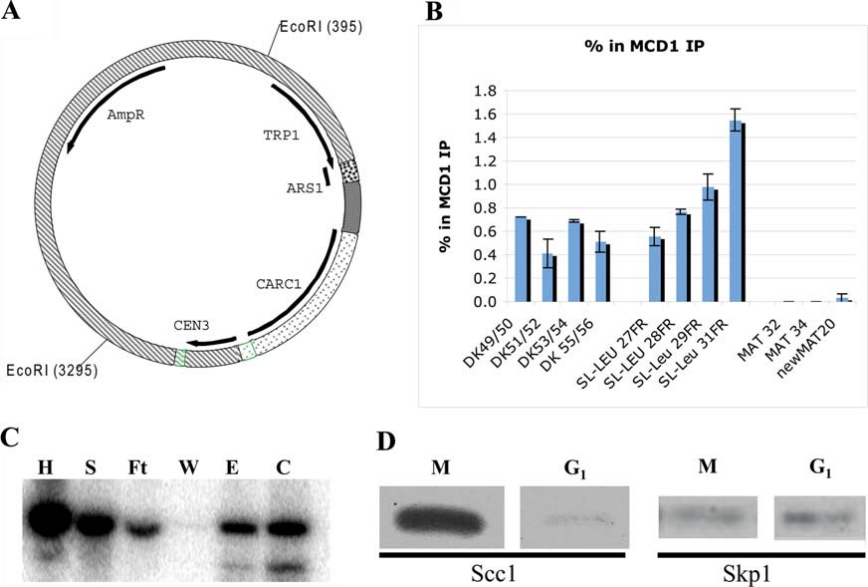
Characterization of pCM26-1. (A) Construct map of pCM26-1. CARC1 and the *CEN3* loci were cloned into the pDTL backbone. Digestion with *EcoRI* removed the bacterial backbone, which included the AmpR gene, while maintaining the *TRP1* and *ARS1* loci. The *lac* operon is located in the ARS region. (B) Chromatin immunoprecipitation of pCM26-1 shows that cohesin binds to the construct. DK49/50 - PCR product over the construct TRP1 locus; DK51/52 - primer product over the 5′ prime end of CARC1; DK53/54 - PCR product over the 3′ prime end of CARC1; DK55/56 - PCR product over the construct CEN3 locus; SL-LEU27R, SL-LEU28R, SL-LEU29R and SL-LEU30R - PCR products over genomic CARC1 (positive control); MAT32, MAT34, newMAT20 - PCR products over genomic MAT locus (negative control). (C) Southern blot of fractions taken during MAP. H – 1% homogenate; S – 1% supernatant (loaded onto column); Ft – 1% flowthrough; W – 5% wash; E – 2% eluate; C-2.5% concentrate. The blot was probed with a *TRP1-ARS1* specific probe. (D) Western blot of samples isolated from nocodazole-arrested cells in M-phase (M) and from alpha-factor arrested cells in G1-phase (G1), with antibodies against either HA tagged Scc1 or Skp1.

In order to study cohesin-chromatin interactions, a minichromosome, pCM26-1 (Figure 2A) was generated that undergoes segregation in a manner similar to chromosomes. pCM26-1 contains the centromeric sequence *CEN3* and CARC1 – an 829 bp CAR located on the left arm of chromosome III, whose 5′ end overlaps with *BUD3*, a non-essential gene involved in bud neck development [17]. The use of both *CEN3* and CARC1 ensured that cohesin enrichment occurred on the minichromosome. Chromatin immunoprecipitation (ChIP) of pCM26-1 showed that 6HA-Scc1p bound to the minichromosome during a nocodazole arrest in M phase (Figure 2B). These ChIP results mirror published data of the *in vivo* binding of cohesin at the CARC1 locus [17]. Directionality of *CEN3* had no bearing on cohesin binding at CARC1 (data not shown). In addition to the *lac* operon that is bound by the *lac-IZ* column during the minichromosome isolation, the plasmid also contains the *TRP1* selection marker and the autonomously replicating sequence *ARS1*, necessary for plasmid replication [22].

The minichromosome affinity purification (MAP) method was previously used to study chromatin structure as well as associated protein complexes, using high-copy number minichromosomes on the order of 30–55 copies per cell [22], [23]. By contrast, pCM26-1 has only 1–2 copies per cell due to the presence of the *CEN3* sequence. Because the previous MAP procedure was not optimized for low-copy plasmids, the yield of the pCM26-1 minichromosome was very low using the published protocol, with less than 0.5% recovery. To overcome the low yield of pCM26-1, it was necessary to optimize the MAP protocol. As described in Materials and Methods, changes were made in the buffer used for the passive diffusion of nuclei, in the salt concentration of the wash buffers, and in the conditions of the concentration step and the sucrose gradient.

To estimate the yield at different steps during the purification process, Southern blot analyses were performed of aliquots taken at each step – homogenate (post-passive diffusion of nuclei incubation), supernatant (sample loaded onto column), high salt wash, eluate, and sucrose gradient concentrate. The results showed that the improved MAP procedure had a 43% recovery of minichromosome from the supernatant, up 100-fold from isolations of this minichromosome using published protocols (Figure 2C). To test whether cohesin was bound to the minichromosomes, western blot analysis of isolated minichromosomes from arrested cells was performed against the 6HA-tagged Scc1p cohesin subunit. Our results show that Scc1p was present in nocodazole arrested samples, but not in alpha-factor arrested samples (Figure 2D), supporting the idea that cohesin was associated with the minichromosome at M-phase but not G1 phase. These results are in excellent agreement with the ChIP and immunoblotting data presented here and previously that show cohesin binding to chromatin [24] and to minichromosomes [25] in M but not G1 phase.

Our results indicate that we have constructed a centromeric minichromosome with a CAR that associated with cohesin in a cell-cycle-regulated manner similar to chromosomal CARs. Furthermore, we have developed a high-yield MAP protocol to allow for the isolation of *in vivo* assembled chromatin-cohesin complexes, which could be used for high-resolution structural analysis via transmission electron microscopy. This modified MAP technique can also be widely applied for the study of other chromatin-protein interactions that are dependent upon normal chromosome segregation. The one caveat to this protocol is that it requires sufficiently high salt during the elution steps and this salt may be disruptive to complexes less stable than cohesin.

### TEM analysis of MAP isolated pCM26-1 shows a circular minichromosome

**Figure 3:**
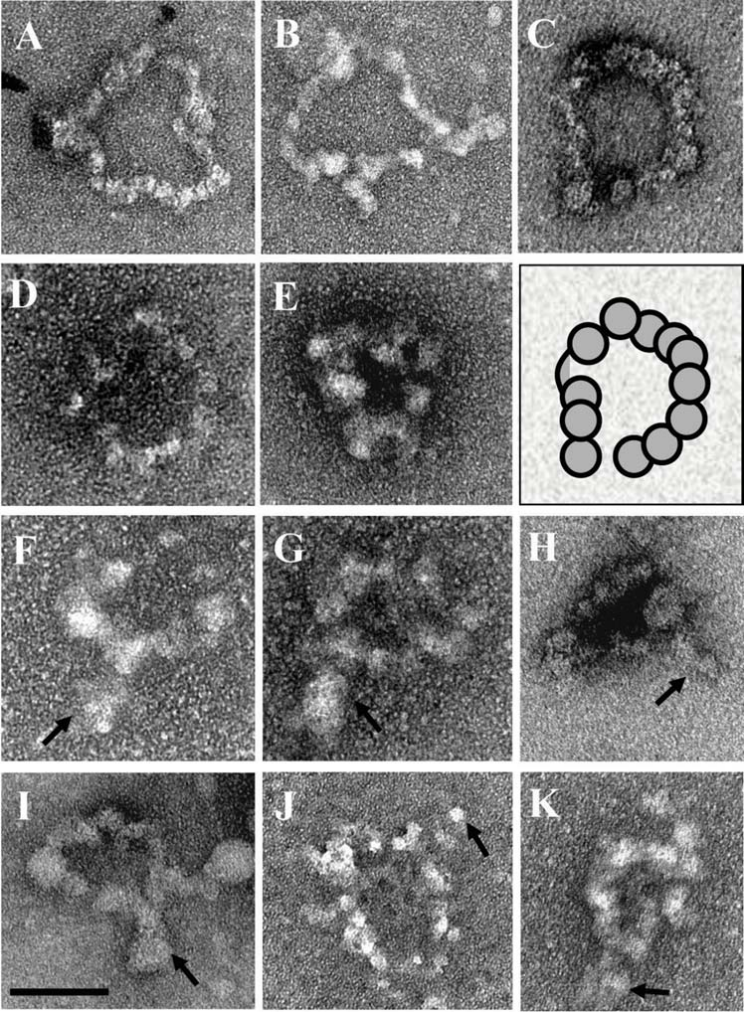
Negatively stained minichromosome pCM26-1 isolated from alpha-factor arrested cells. All minichromosome samples from alpha-factor arrested cells were identified as continuous stretches of chromatin characterized by a closed circular shape with beads-on-a-string morphology. The diameter of these samples was measured to be 60nm±4.3nm (n = 16). Diameter measurements of nucleosomes showed them to be 10.6nm±1.4 (n = 38). The schematic panel is a 1∶1 diagram of the sample in panel C. The nucleosomes are shown as circles. A subset of minichromosomes exhibited a small protrusion of varying length. This structure is shown in panels F–H and is indicated by the black arrows. Scale bar = 50nm.

**Figure 4:**
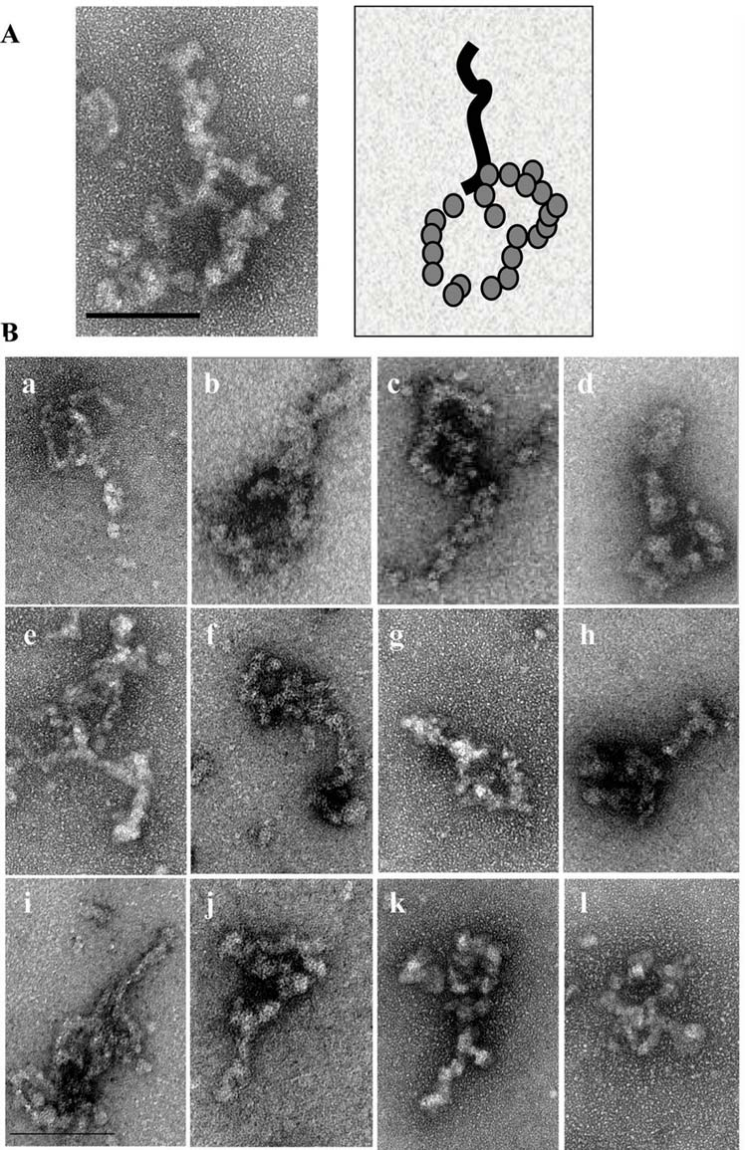
Negatively stained pCM26-1 isolated from nocodazole-arrested cells. (A) Image on the left shows two replicated minichromosomes interacting at one end of a long flexible rod protruding from the minichromosomes. The panel on the right is a schematic of the image. Aqua circles represent nucleosomes of ∼10nm in diameter on chromatin rings ∼60nm in diameter. Scale bar = 100nm. (B) Samples are negatively stained and were identified by the presence of two minichromosomes of the same morphology as those observed from alpha-factor arrested cells. Panels b, c, and k show twisting along the length of the protruding rod. Kinks in the rod can be seen in panels e and f. Panel i shows bifurcation at the end of the rod closest to the minichromosomes. Scale bar = 100nm.

To ascertain whether the MAP preparations yielded intact minichromosomes that could be used to obtain structural information, samples were fixed, stained, and examined by TEM. Samples were prepared from both G1 phase arrested cells when cohesin is not bound to centromeric regions or CARs and from M phase arrested cells when cohesin is bound to centromeric regions and CARs. In previous studies, minichromosomes have been documented as continuous stretches of chromatin, with the characteristic beads-on-a-string morphology indicative of nucleosomes [22], [23]. This morphology has also been observed in TEM analyses of chromatin samples and nucleosome arrays (see [26]–[28] for examples). Similarly, in both samples from G1 and M phase cells, pCM26-1 was identified as a circular expanse of chromatin with variable flexibility (Figures 3 and 4). Of the samples imaged, sixteen were from alpha factor arrested G1 phase cells and thirty-one were from nocodazole arrested M phase cells. Minichromosomes exhibited circular configurations (Figure 3, C–E), general oval configurations (Figure 3, A–B), and circular configurations with extensions of variable length, from 10–20nm (Figure 3, F–H). The minichromosomes were distinct from other material that adhered to the grids, mainly viruses and genomic contaminants, which were differentiated respectively, by their smooth surface and non-circular appearance (Figure S1).

To further characterize the structure of pCM26-1, several measurements were taken – the diameter of the minichromosome and the diameter of the beads on a string, considered to be nucleosomes. The average diameter of the minichromosomes from G1 cells was 60nm±4.3nm (n = 16) and from M phase cells 56nm±2.6nm (n = 64). Minichromosome images from G1 cells with short extensions had a diameter of 51.3nm±5.1nm, smaller than minichromosomes without an extension. Closer examination of these protrusions, which occurred in approximately 30% of the images taken from G1 arrested samples, showed that when the length of the protrusion was taken into account, the diameter of the minichromosomes calculated from circumference measurements increases from 51.3 to 62.4nm, in keeping with the diameter measurements from samples that did not exhibit protrusions. The somewhat variable shape of the minichromosomes suggests that they are relatively flexible; therefore, it is likely that some minichromosomes were folded over during sample preparation, resulting in images showing a short extension and a smaller measureable diameter. Examples of how measurements were taken are shown in Figure S2.

The minichromosome diameter measurements were in keeping with minichromosome sizes of other constructs isolated by MAP and imaged by TEM [22], [23]. Measurements of the nucleosome diameters from G1 cells yielded an average of 10.6±1.4nm (n = 38) and 10nm±1.5nm (n = 72) from M phase cells. These measurements were in agreement with the nucleosome published diameter of 11nm [29]. From the overall morphology and these measurements, we concluded that we had purified minichromosomes and obtained representative TEM images from both G1 and M phase cells.

### TEM analysis from M-phase cells shows replicated minichromosomes associated at one end of a flexible rod

While the minichromosomes from G1 cells appeared as singular circles consistent with them being separate unreplicated plasmids, minichromosomes from M phase cells were always observed as two connected circles, representing still-attached replicated plasmids. The constraint placed on identifying replicated minichromosomes was that the plasmids had to be within three cohesin lengths of each other. The length of the cohesin heterodimer was identified by previous TEM studies to be 64±6nm [13]. Minichromosomes from M phase cells were always seen in close proximity to each other, exhibiting partial overlap in some of the images. Only one singular minichromosome was seen in the samples prepared from M phase arrested cells. This occurrence was consistent with that fact that nocodazole arrests 90–95% of cells in a given culture at M phase, allowing some cells to progress to G1.

When we examined the minichromosomes from M phase cells, we noticed a striking feature: in addition to two adjacent circles representing two replicated minichromosomes, there was a prominent rod-like structure with one end at or near the junction between the minichromosomes, forming a “closed-scissors conformation” (Figures 4, A–B). This was true for each of the 32 double-circle images we observed. In some images, the two replicated minichromosomes were separate but next to each other (Figures 4B, c,e,f,i,j,l); in some, they seemed to partially overlap, presumably when they adhered to the grid (Figure 4B, a,b,d,g,h,k). In addition, the rod-like structure in each image of the replicated minichromosomes was much longer than the extensions of variable length seen in some of the G1 minichromosome images. While this rod appeared straight in most images, some had a bend or kink along the length of the rod, suggesting that they were somewhat flexible (Figure 4B, c,e,f). In addition, a bifurcation of the rod at the end near the minichromosomes can be seen in at least one image (Figure 4Bi). This type of closed-scissors image was neither detected among G1 phase minichromosomes, nor in previous studies of minichromosomes that lacked cohesin-binding sequences [22], [23].

Because the pCM26-1 minichromosome contains *CEN3* unlike previous minichromosomes studied using MAP and TEM, it is possible that the rod described above represented a centromere/kinetochore structure. It is unlikely that the entire kinetochore was retained in the minichromosome purification because the salt concentration of the elution buffer during the MAP experiments (250mM NaCl) was high enough to disrupt kinetochore-chromatin interactions [30]. However, the kinetochore scaffold complex centromere binding factor 3, CBF3, might be stable enough to remain associated with the M and G1 phase minichromosomes after purification. To further examine this possibility, we performed western blot analyses of the minichromosome fractions from M and G1 arrested cells using an antibody to Skp1, a component of CBF3. Our results from this western blot indicate that Skp1 is present in samples isolated from both M and G1 phase arrested cells (Figure 2), suggesting that CBF3 is present in both minichromosome preparations.

The TEM images clearly showed that most G1 phase minichromosomes lacked protrusions, and only about one third had protrusions with variable lengths that were consistent with the images representing folded circles, as supported by the detailed measurements described above. In contrast, M phase minichromosomes all had a much longer rod shaped structure. Therefore, the presence of CBF3 in both G1 and M phage minichromosome samples suggests that CBF3 or kinetochore cannot explain the rod shaped structures found only in the M phase minichromosomes. Additionally, western blot experiments of isolated minichromosome samples detected the cohesin complex from M phase cells but not G1 cells (Figure 2D). Therefore, our ChIP, western, and TEM results combined to support the conclusion that the rod shaped structures associated with M phase minichromosomes most likely represent cohesin complexes. Nevertheless, we recognize the unlikely possibility that the rod shaped structures might be related to the kinetochore; if true, this would represent the first direct imaging of the budding yeast kinetochore complex.

To characterize the rod structures further, we took several measurements of each rod in the 32 images. In addition to the measurements of the individual minichromosome mentioned above, the length the rod was also measured and found to be 70±11nm. Measurements of the length of the rod are consistent with other measurements of the cohesin holocomplex (64±6nm) and slightly longer than published measurements of the Smc1/Smc3 heterodimer (59±4nm) [12], [13]. Examples of how measurements were taken are shown in Figure S2C. From the length of the rod structure and its cell cycle dependent appearance, the most plausible explanation is that it is cohesin. This explanation would make these images the first of *in vivo* assembled cohesin-chromatin complexes.

Our observations of the TEM images strongly support the idea that the replicated minichromosomes are restricted to one end of the extended rod. These images of the “closed scissors” confirmation showing two replicated minichromosomes constrained by a possible cohesin rod are surprising and not predicted by proposed models for cohesin binding (Figure 1B). Because the embrace or extended-embrace models do not limit the interaction of the topologically trapped minichromosomes within the cohesin ring to a specific cohesin domain, we would anticipate that the two replicated minichromosomes would be distributed at many sites along the circumference of an open ring. Nevertheless, the idea that cohesin encircles the two minichromosomes as proposed in the embrace model could be modified to include interactions between the Smc1 and Smc3 coiled-coil domains. This added interaction would restrict the position of the minichromosomes to a specific region of cohesin. This revision to the embrace model with additional interactions between Smc1 and Smc3 and between cohesin and chromatin could account for the TEM observations. In the snap model, the two sister minichromosomes are each bound to an end of individual cohesin molecules, which are additionally tethered to each other. With this model, we would expect to see a separation between the minichromosomes of two cohesin lengths or more.

Of the models previously published in the cohesin literature, the physical interaction model is most consistent with our results, because it proposes the binding of sister chromatids to a specific position in the cohesin complex. In addition to studies on the condensin complex, it has been reported that Smc1 and Smc3 C-terminal fragments are capable of *in vitro* binding of DNA [31]. Recent results from FRET analyses suggest that Smc1 and Smc3 head domains are in close proximity, even in the absence of Scc1 [15]. This is consistent with both of the head domains interacting with the same cohesin-binding site. The TEM images did not reveal which end of cohesin was bound to the minichromsomes. However, based on the DNA binding data for both condensin and the condensin-like bacterial mukB complexes which demonstrate tight DNA-SMC head interactions, as well as imaging of the condensin complex to DNA which reveals the association of the head domain to DNA, it is possible that the Smc1 and Smc3 heads bind to chromatin [20], [32], [33].

### Width measurements of the rod imply coiled-coil interactions between multiple cohesin molecules

In addition to length measurements of the rod, we took measurements of the width of the rod and found the width to be 14±4 nm. Previous reports of similarly stained antiparallel coiled-coil domains, such as the stalk from dynein, demonstrate a width of approximately 2nm [34]. Therefore, the observed rod width was significantly greater than the expected width of a single coiled-coil domain, such as that of Smc1, or two coiled-coil arms, such as those of Smc1 and Smc3. The width of 14±4 nm is sufficient to account for 6–8 SMC arms or 3–4 cohesin holocomplexes per pair of minichromosomes, if each holocomplex contains one pair of heterodimeric Smc1/Smc3 proteins.

The inferred presence of multiple cohesin holocomplexes appearing as a rod structure at a single locus raises the possibility of intermolecular interactions between the arms of the cohesin molecules. This type of tetrameric or higher-order interaction of coiled-coil arms is not novel as it forms the basis of, among others, myosin filament assembly and the tetramers of influenza hemogglutinin HA2 [35]–[39]. Furthermore, a number of *in vivo* and *in vitro* observations are consistent with a role for cohesin arms in cohesin function beyond serving just as spacers between two active domains. The amino acid composition of the arm domains within vertebrate Smc1 and Smc3 shows low divergence, suggesting a function for the arm residues beyond simple coiled-coil formation [40]. Additionally, five residue insertions within one cohesin arm disrupted cohesin function, indicating that the cohesin arm is essential for cohesin binding and for cell viability [18]. The suggestion that *in vivo* assembled, chromatin-bound cohesin may form a multimeric rod structure is in keeping with numerous observations from previous biochemical studies [12]–[15]. For example, cleavage at one of several points within the cohesin coiled-coil domains facilitated by insertions of Tobacco Etch Virus (TEV) protease sites leads to cohesin-chromatin dissociation [14]. While these findings were previously used to support the notion that cohesin forms an open ring whose cleavage leads to sister chromatid dissociation, it is equally likely that cleavage along the coiled-coil domains affects intermolecular interactions along the SMC arms necessary for cohesion. In addition, recent FRET analyses did not detect energy transfer betwee two Smc1 heads or between two Smc3 heads [15]. Because the maximal distance detectable by FRET is 10 nm, with an even shorter optimal distance, the Smc1 (Smc3) heads could still be spaced with a distance of 10 nm or slighter longer, compatible with the width of the rod from the TEM results.

### Conclusions

We report the adaptation of a high-yield purification technique for isolating low-copy centromeric minichromosomes from the budding yeast. This MAP technique can be useful for analyses with minichromosome systems that are low copy, such as those requiring centromeric function. Additionally, we report the first direct imaging of structure that most likely represents *in vivo* assembled cohesin complexes on replicated sister minichromosomes. Our ChIP, western, and TEM results together strongly support the idea that cohesin forms a rod structure and that minichromosomes interact at one end of this rod, in a manner not predicted by the embrace, extended embrace, and snap models. Additionally, width measurements of the rod suggest that sister chromatid cohesion might be mediated by multiple cohesin molecules that interact with each other along their coiled-coil arms. As detailed above, our results are consistent with the body of published data characterizing the *in vitro* interactions of the components of the cohesin complex. Although our data do not rule out the embrace or the physical interaction models for cohesin *binding* to chromatin (Figure 1), the TEM images suggest that the models are likely not accurate representations of cohesin *maintenance* at CAR loci. For example, one can envision a singular cohesin ring opening and closing around sister chromatids at CAR loci, as has been previously suggested [42]. The presence of additional rings, however, may trigger an intermolecular interaction along the arms of neighboring cohesin complexes, leading to “bundling” of the rings and narrowing the localization of the SCC to a short region, defined by CARs. Similarly, the heads of the SMC proteins from one cohesin ring may interact intermolecularly with the SMC heads from another (as has been proposed in the bracelet model by Huang et al.), bringing the arms in close enough proximity for them to interact and collapse [43]. The images obtained from using the MAP technique give a post-replication snapshot of probable cohesin-chromatin interactions at CAR loci. Our results provide crucial structural information that is potentially valuable for the interpretation of future functional studies and that may facilitate a better understanding of the mechanisms of cohesin-chromatin interactions.

## Materials and Methods

### Strains and Plasmids

All cloning work and plasmid purifications were done in the *Escherichia coli* DH5α strain. The *lac-IZ* fusion protein was expressed from pTLIZ plasmid in *Escherichia coli* BL21-DE3 cells [22]. All yeast strains were isogenic with strain W303 (*MATa/MATα ADE2/ade2 CAN1/can1-100 CYH2/cyh2 his3-11,15/his3-11,15 LEU1/leu1-c LEU2/leu2-3,112 trp1-1:URA3:trp1-3*′*/trp1-1 ura3-1/ura3-1*). Strain 8803 (6HA-Mcd1p) was generously provided by Frank Uhlmann (described in [41]). The 800 bp region spanning CARC1 was amplified by PCR and cloned into the pUNI vector of the Echo Cloning System (Invitrogen, http://www.invitrogen.com). The fragment was removed upon double digest with *Sac1* and *Not1* and cloned into the pDTL backbone [22]. A 470bp *Sac1* fragment containing the 300bp *CEN3* and flanking vector sequence was subsequently cloned into the *Sac1* site of pDTL/CARC1. All minichromosomes were digested with *EcoRI* to remove the bacterial backbone and religated prior to transformation into yeast strains. Transformation was verified by Southern blot analysis.

### Yeast Cell Growth and Synchronization

Yeast cells were grown exponentially in appropriate minimal media for maintaining the plasmids with 20% dextrose at 30°C, with aeration by shaking. Cells were arrested at the G1 or M phase by the addition to a final concentration of 10mg/ml alpha factor (Proteomics and Mass Spectrometry Facility, Penn State College of Medicine, Hershey Park) or 15 µg/ml nocodazole (USBiological, Swampscott) respectively as previously described [17]. Cell synchronization was monitored by propidium-iodine (Sigma-Aldrich, St Louis) staining flow cytometry analysis on a Beckman-Coulter XL-MCL I single laser cytometer (Miami).

### Chromatin Immunoprecipitation

ChIP experiments were completed as performed by (Unal *et al*., 2007).

### Minichromosome Affinity Purification (MAP)

MAP was adapted from the previously described procedure [22]. Cells usually were grown in a 4L culture and arrested at the derived phase of the cell cycle. Harvested cells were washed two times in 30 ml SB (1.4M sorbitol, 40 mM HEPES, 0.5 mM MgCl_2_, pH 7.5) with 1 mM phenylmethylsulfonyl fluoride (PMSF, Sigma-Aldrich) and 10 mM β-mercaptoethanol (Fisher Scientific, Pittsburgh) and pelleted by centrifuged at 5,000 rpm in an SS34 or Sorvall G-20 for five minutes. Cells were resuspended in SB, 1 mM PMSF to a total volume equivalent to 4× wet pellet weight. To partially digest the yeast cell walls, 10mg/ml freshly made zymolyase (Associates of Cape Cod, East Falmouth) was added to a final concentration of 0.5mg/ml and the sample was incubated at 30°C for approximately 20 minutes or until spheroplasting was completed, as determined microscopically. Volume was brought up to 30 ml SB, 1 mM PMSF and the sample was centrifgued at 5,000 rpm for 5 minutes. All subsequent steps were performed on ice.

Pellets were gently resuspended with plastic 25 ml pipettes and washed twice in 30 ml cold SB, 1 mM PMSF, upon centrifugation at 5,000 rpm in an SS34 or Sorvall G-20 rotor for five minutes. Pellets were resuspended in 10 ml MBB (150 mM NaCl, 20 mM HEPES, 1 mM EDTA, 0.6% Tween-20, pH 8.0), with 1 mM PMSF, 10 µg/ml A-protinin, 2 µg/ml leupeptin, 2 µg/ml pepstatin A (Sigma-Aldrich) and incubated on ice for 15 minutes. The chilled spheroplasts were lysed in a Thomas glass homogenizer and Teflon motor-driven pestle (Swedesboro) with 8 strokes. Samples were incubated on ice for 3–4 hours with gentle agitation on a platform shaker to allow for the passive diffusion of minichromosome from the nuclei, then centrifuged at 18,000 rpm for 30 minutes to precipitate cellular debris. The supernatant was incubated on a rotator with column resin charged with *lac-IZ* fusion protein (see Ducker *et al*., 2000 for column preparation) at 4°C for one hour, before being loaded onto the column by gravity at a flow rate of 0.5ml/minute. The column was washed with 20 ml MBB, followed by 20 ml MBB-200 (MBB with 200 mM NaCl). 1 ml elution buffer, EB (300 mM NaCl, 20 mM HEPES, 1 mM EDTA, 0.1% Tween-20, pH 8.0) was applied to the column and allowed to flowthrough. An additional 1 ml EB was added and was incubated on the column for 30 minutes. An additional 3 ml EB were added to the column and the eluate was collected in its entirety and diluted immediately 1∶1 in cold distilled water.

The diluted eluates were concentrated by centrifugation in Nanosep 30K omega columns (Pall Co., East Hills) to a final volume of 300–400 µl. The concentrated eluates were loaded onto 15–40% sucrose gradients (Ultrapure sucrose, Gibco BRL, Carlsbad) and spun at 40K, 4°C for 4 hours. Samples were harvested in 0.5 ml aliquots, of which 25 µl was proteinase K treated at 50°C for 2 hours before being loaded onto a 0.8% TBE agarose gel (SeaKem ME), transferred to Hybond-NX membrane (Amersham-Biosciences), and assessed by Southern blot analysis with a probe specific to TRP1-ARS1. Sucrose gradient aliquots containing minichromosome were centrifuged in Nanosep 30K concentrators (Pall Gelman Lab), with a volume reduction to 50–100 µl. To monitor recovery of sample throughout MAP, 1–5% of sample was removed at the following steps: after passive diffusion of nuclei (1%), prior to application to the column matrix (1%), flowthrough off the column (1%), wash (5%), eluate (2%), and concentrate from sucrose gradient (2.5%). These samples were treated with 100 µg/ml RNaseA (Sigma-Aldrich) at 37°C for 30–120 minutes. 2 µl proteinase K was added in addition to SDS to a final concentration of 1%. Samples were incubated at 50°C for a minimum of 2 hours, phenol chloroform extracted, and ethanol precipitated. Precipitates were resuspended in 20 µl 0.1XTE and recovery was monitored by gel electrophoresis and Southern blot analysis as previously described. Band intensities were quantified using the Image Quant software program.

### Transmission Electron Microscopy Sample Preparation and Analysis

Minichromosome samples concentrated from the MAP sucrose gradients were dialyzed against HEN10 buffer (10 mM NaCl, 10 mM HEPES pH 7.5, 1 mM EDTA) in a Slide-A-Lyzer MINI dialysis unit (Pierce, Rockford) at 4°C overnight. Samples were fixed by dialysis against HEN buffer with 1% gluteraldehyde (Electron Microscopy Sciences) for 4–6 hours at 4°C. Excess gluteraldehyde was removed by dialysis against HEN buffer at 4°C for at least 4 hours. A 5 µl sample drop was diluted 1∶1 with HEN100 (100 mM NaCl, 10 mM HEPES pH 7.5, 1 mM EDTA) buffer on parafilm. A carbon-coated 400 mesh copper grid (Spi Supplies, Inc) glow discharged for 2 minutes was floated on the sample drop for 10 minutes at 22°C. Excess solution was removed by touching the grid to the edge of Whatman paper. The grid was washed three times for 15 seconds with HEN50 (50 mM NaCl, 10 mM HEPES pH 7.5, 1 mM EDTA). For positive staining with aqueous uranyl acetate (UA), the grid was placed on three successive drops of 2% UA for 30 seconds on each drop. The grid was then washed with water for 30 seconds per drop. After the last drop, the grid was air-dried overnight. For negative staining with UA, the grid was washed as above after adhesion, and then stained on three successive drops of 2% UA for 30 seconds per drop. Grids were viewed with a JEOL 1200 Ex-II Transmission Electron Microscope (Peabody) and pictures were taken on a TIETZ camera (Gauting, Germany).

### Western blot analysis

Samples from various stages of the MAP experiments were loaded onto a 12% SDS-polyacrylamide gel and run at 120V for 1.5–2 hours. Equivalent amounts of minichromosomes were added from isolated samples as determined by EtBr staining and ImageQuant analysis of samples from G1 and M phase arrested cells. The samples were transferred onto Immuno-blot PVDF membrane (Bio-Rad, Hercules) in transfer buffer (192 mM glycine; 25 mM Tris-HCl) overnight at 30V. Blots were blocked for one hour at room temperature in 1% non-fat milk/PBS-T (1XPBS, 0.1% tween). After being rinsed twice with PBS-T for fifteen minutes per wash, the blots were incubated at room temperature for one hour with primary antibody (HA, 1∶5000, from Covance, Skp1 1∶500, from Santa Cruz Biotech) in 0.5% non-fat milk in PBS-T and then rinsed again, twice with PBS-T. The blots were incubated with a secondary antibody (goat anti-mouse, 1∶2000, from Covance) for one hour. After the final 2×15 minute washes in PBS-T, the blots were incubated with pico-chemilluminesce reagents (Biorad) for 2 minutes and exposed to film (Kodak) for 2 minutes to overnight to obtain optimal exposure.

## Supporting Information

Figure S1 Negatively stained non-minichromosome material visible in TEM analysis. (A) Viral material distinguishable from minichromosomes due to their smooth surface. Scale bar = 50nm. (B) Dust and dirt particles distinguishable from minichromsomes due to their irregular shapes. Scale bar = 100nm. (C) Genomic contaminent containing the same beads-on-a-string morphology as the minichromosomes, but without the circular appearance of the minichromosomes. Scale bar = 100nm.
(0.63 MB TIF)

Figure S2 Sample measurements taken of images from G1 and M phase arrested samples. Measurements were taken with the program GIMP as indicated in the Materials and Methods section. (A) Singular minichromosome from G1 arrested cells. Multiple measurements of the diameter of each minichromosome were taken (solid lines), as is the diameter of identifiable nucleosomes (dashed lines). (B) Singular minichromosome with extension from G1 arrested cells. Multiple measurements of the diameter of each minichromosome were taken (solid lines), nucleosome diameters (short dashed lines), and the length of the extension (long dashed line). The minichromosome diameter measurements were used to calculate the circumference of the minichromosome (πd, where d is the diameter), to which was added the length of the extension twice. This resulted in an overall estimate of the true circumference and diameter of the minichromosome. (C) Replicated minichromosomes with rod-shaped structure. Multiple measurements were taken of the diameter of the minichromosomes (solid black lines), the length of the rod structure (dashed lines), and the width of the rod structure (solid gray lines).
(2.44 MB TIF)
