# Supplementary figures and images for: Cohesin Interaction with Centromeric Minichromosomes Shows a Multi-Complex Rod-Shaped Structure
Source: PLoS One. 2008 Jun 11;3(6):e2453. doi: 10.1371/journal.pone.0002453 (PMC2408725; doi:10.1371/journal.pone.0002453)

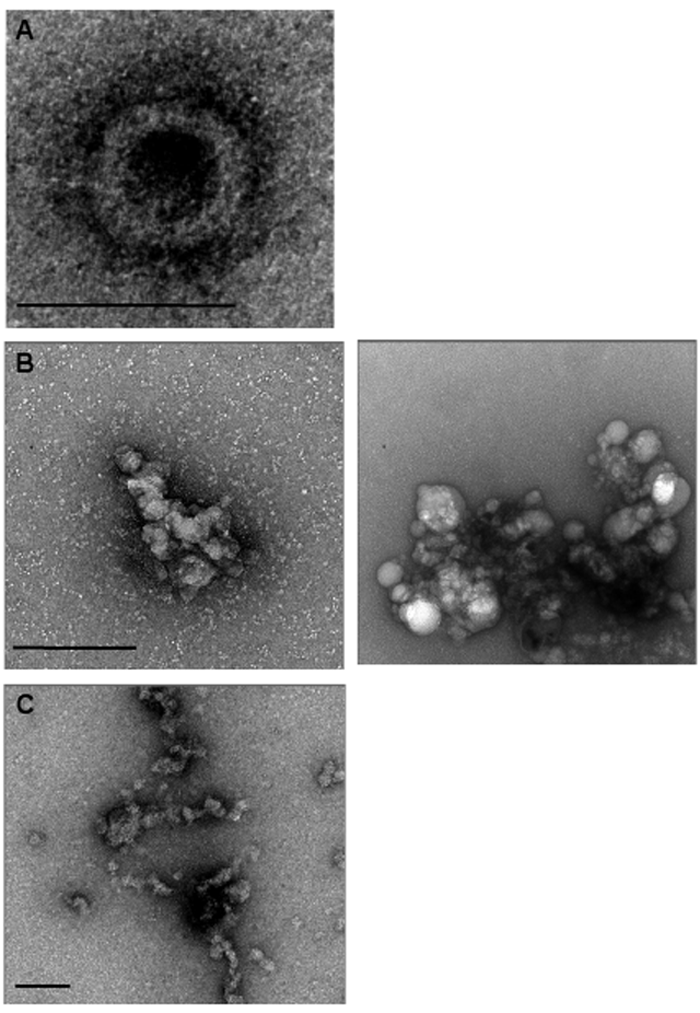

Supplement: Figure S1 — Negatively stained non-minichromosome material visible in TEM analysis. (A) Viral material distinguishable from minichromosomes due to their smooth surface. Scale bar = 50nm. (B) Dust and dirt particles distinguishable from minichromsomes due to their irregular shapes. Scale bar = 100nm. (C) Genomic contaminent containing the same beads-on-a-string morphology as the minichromosomes, but without the circular appearance of the minichromosomes. Scale bar = 100nm. (0.63 MB TIF) [file pone.0002453.s001.tif]

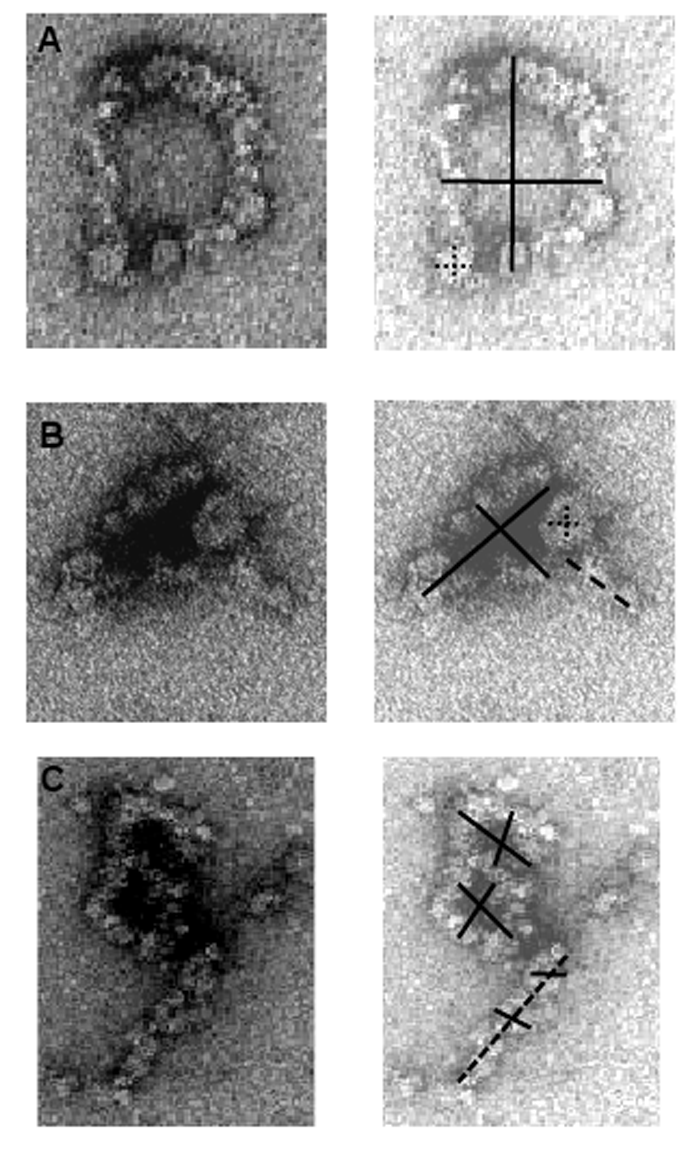

Supplement: Figure S2 — Sample measurements taken of images from G1 and M phase arrested samples. Measurements were taken with the program GIMP as indicated in the Materials and Methods section. (A) Singular minichromosome from G1 arrested cells. Multiple measurements of the diameter of each minichromosome were taken (solid lines), as is the diameter of identifiable nucleosomes (dashed lines). (B) Singular minichromosome with extension from G1 arrested cells. Multiple measurements of the diameter of each minichromosome were taken (solid lines), nucleosome diameters (short dashed lines), and the length of the extension (long dashed line). The minichromosome diameter measurements were used to calculate the circumference of the minichromosome (πd, where d is the diameter), to which was added the length of the extension twice. This resulted in an overall estimate of the true circumference and diameter of the minichromosome. (C) Replicated minichromosomes with rod-shaped structure. Multiple measurements were taken of the diameter of the minichromosomes (solid black lines), the length of the rod structure (dashed lines), and the width of the rod structure (solid gray lines). (2.44 MB TIF) [file pone.0002453.s002.tif]
